# Triggering hydrogenolysis of the lignin model compound benzyl phenyl ether using the intrinsic exothermicity of Pd-hydride formation

**DOI:** 10.1039/d5su00574d

**Published:** 2025-10-08

**Authors:** Erin V. Phillips, Marta C. Hatzell, Carsten Sievers

**Affiliations:** a School of Chemistry and Biochemistry and School of Chemical and Biomolecular Engineering, Georgia Institute of Technology Atlanta Georgia 30332 USA carsten.sievers@chbe.gatech.edu; b School of Chemical & Biomolecular Engineering, Georgia Institute of Technology Atlanta Georgia 30332 USA; c George W. Woodruff School of Mechanical Engineering Atlanta Georgia 30318 USA

## Abstract

When supported Pd catalysts are exposed to hydrogen, the heat released from the spontaneous and exothermic Pd-hydride formation creates a reaction environment that allows for melting and hydrogenolysis of benzyl phenyl ether (BPE) under nominally ambient conditions. The intrinsic exothermicity of this hydride formation acts as an initiating force for α-O-4 ether cleavage of the BPE dimer, without the need for external heat to be applied to the reaction system. Thermogravimetric analysis with differential scanning calorimetry shows heat flows of 58, 40, and 32 W g^−1^ for Pd supported on carbon, silica and alumina, respectively. BPE conversion increased with increasing heat flow, which correlated with a higher Pd particle dispersion and lower heat capacity of the support. X-ray absorption spectroscopy at the Pd-K edge confirms Pd-hydride formation. This work shows that the heat released by Pd-H formation can be used as an initiator for α-O-4 ether cleavage in a solid lignin model compound.

## Abstract 2

When Pd catalysts are exposed to H_2_, the heat released from the spontaneous, exothermic Pd-hydride formation creates a reaction environment that allows for melting and hydrogenolysis of benzyl phenyl ether under nominally ambient conditions.

## Graphical abstract

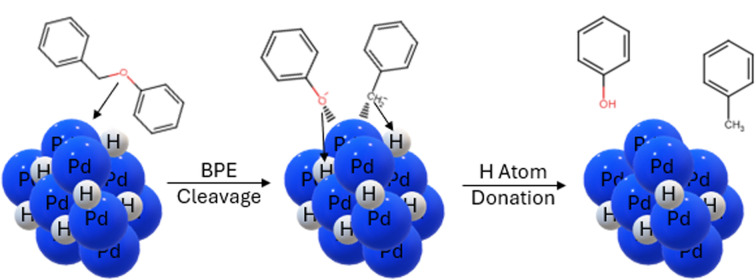

**Sustainability spotlight**

Processing solid feedstocks over heterogeneous catalysts typically requires that either the feedstock or the catalysts are brought into a liquid form to achieve contact on a molecular scale. This typically involves the use of significant quantities of solvents, which are often toxic. Our work presents an approach to initiate hydrogenolysis of benzyl phenyl ether, a lignin model compound, by melting it using the heat released from converting supported palladium catalysts to their hydride form. High conversion can be reached within minutes with gentle agitation as the only form of external energy supplied. The work aligns well with UN Sustainable Development Goal 12 – Responsible Consumption and Production and others that imply the reduction of environmental pollution.

## Introduction

For decades, palladium-based catalysts have been used for hydrogenation and hydrogenolysis reactions.^1–24^ Pd-hydrides readily form when Pd is exposed to H_2_ under ambient conditions,^25^ with a 2 : 3 ratio of H : Pd at standard temperature and pressure (STP) and an exothermic enthalpy of reaction of about −40 kJ mol^−1^.^26^ Many studies have been published on favorable sites of Pd-H formation and the energetics of surface adsorption and subsurface hydrogen absorption.^27–32^

This phenomenon of membrane diffusion is unique to palladium, where H_2_ atoms readily dissociate into H atoms and diffuse into the particle.^33–35^ As Pd lattice vacancies are filled by H atoms, the lattice expands, and more vacancies are created.^31^ As a result, lattice expansion causes the number of sorption sites to continually increase during Pd-H formation. This expansion enhances surface adsorption, which is more exothermic than subsurface adsorption.^31^ Because a lower energetic input is required for surface interactions between hydrogen and palladium, surface adsorption becomes the predominant and more favorable interaction.^36^ In turn, this facilitates the participation of Pd-H surface species in catalytic reactions.^28,31^ By contrast, subsurface adsorption requires significant lattice restructuring, making it inherently less exothermic.^28,31^ However, in a reactive system, the hydrogen atoms adsorbed on the surface of the Pd particle can continuously diffuse into the particle. As a result, an equilibrium between surface and subsurface hydride saturation is approached.^28,31^ Therefore, once a catalytic reaction reaches completion, and surface hydrides are no longer actively being consumed, diffusion into the particle subsurface occurs.

Pd catalysts are commonly used in hydrogenation and hydrogenolysis reactions because of their high activity and selectivity. Because Pd hydrides readily form, these catalysts act as a hydrogen reservoir and are able to donate an H atom when in contact with the feedstock. Mechanocatalytic systems are optimal for this type of H atom donation, because milling is conducted in a solventless environment, so the catalyst and feedstock are consistently in direct contact within the milling vessel. Many studies of lignin conversion have examined hydrogenolysis of model compounds with ether bonds such as benzyl phenyl ether (BPE, α-O-4 bond),^37–56^ diphenyl ether (DPE, 4-O-5 bond)^38,43,48,49,51,55,57–63^ and phenethoxybenzene (PEB, β-O-4 bond) over Pd catalysts.^38,43,48,49,51,53–55,64–72^ Most of these studies used autoclave reactors and elevated temperatures and pressures.

This study investigates the ether cleavage of the α-O-4 bond in BPE using the heat released from the spontaneous exothermic formation of Pd-H as a sufficient energy source to initiate hydrogenolysis. The three Pd catalysts that were investigated with this work were supported on carbon, silica and alumina. The reactions were performed in a ball mill vessel, which was adapted for H_2_ gas flow with low frequency mixing at 3 Hz, under ambient temperatures and pressures. The results demonstrate that when a sufficient amount of catalyst is used, full conversion and high carbon efficiencies are attainable, within 15 min or less.

## Results and discussion

Inductively coupled plasma (ICP) was used to determine the exact weight loading of the three supported Pd catalysts that were used experimentally in this study. Results showed 4.07%, 3.84% and 3.62 wt% for Pd on C, Pd on SiO_2_, and Pd on Al_2_O_3_, respectively. The catalysts were denoted Pd_04_/C, Pd_04_/SiO_2_ and Pd_04_/Al_2_O_3_ accordingly. Transmission electron microscopy (TEM) (S1.4.2, eqn (S3)) showed that Pd_04_/C had an average initial particle size of 2.5 nm and a 45% dispersion. For Pd_04_/SiO_2_, the metal particles measured 6.3 nm in diameter (18% dispersion), while Pd_04_/Al_2_O_3_ had Pd particles with an average diameter of 3.2 nm (35% dispersion).

**Fig. 1:**
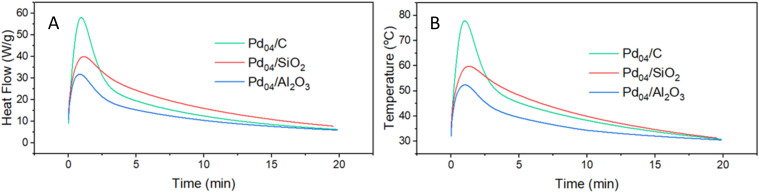
TGA/DSC of Pd_04_/C, Pd_04_/SiO_2_ and Pd_04_/Al_2_O_3_. Catalysts (7.5 mg) were exposed to H_2_ at 10 mL min^−1^ for 20 min with no additional external heating. Shown is heat flow in W g^−1^ (A) and temperature (B) to elucidate the heat generated by intrinsic and spontaneous Pd-hydride formation.

Differential scanning calorimetry (TGA/DSC) was used to determine heat flow as a result of hydride formation. For this purpose, 7.5 mg of the catalysts were added to a crucible, and placed in a thermogravimetric analysis-differential scanning calorimetry (TGA/DSC) instrument and purged with N_2_. The reaction started by switching the gas flow to H_2_ at 10 mL min^−1^ at 20 °C (Fig. 1). Within 2 min, the heat flow of all three catalysts peaked, indicating that spontaneous Pd-H formation occurred immediately upon H_2_ exposure. Pd_04_/C reached a maximum heat flow of 58 W g^−1^, Pd_04_/SiO_2_ reached 40 W g^−1^, and Pd_04_/Al_2_O_3_ reached 32 W g^−1^ (Fig. 1A). The change in temperature was 58 °C for Pd_04_/C, 40 °C for Pd_04_/SiO_2_ and 32 °C for Pd_04_/Al_2_O_3_ (Fig. 1B). For Pd_04_/SiO_2_ and Pd_04_/Al_2_O_3_, these values fell within the range of the theoretical values calculated based on Pd content of the catalyst and heat capacity for each support (Fig. S1 and S2, eqn (S5)). For Pd/C, up to 5 wt% hydrogen can be adsorbed on the surface of graphitic materials.^73^ This adsorption is exothermic and contributes to the heat release. For this reason, Pd_04_/C had a significantly higher heat flow when compared to Pd_04_/SiO_2_, even though they have similar heat capacities. Additionally, the heat flow of Pd_04_/SiO_2_ decreased less rapidly when compared to Pd_04_/C and Pd_04_/Al_2_O_3_ because the Pd particles on SiO_2_ were larger, so subsurface hydride diffusion occurred more slowly.

Based on these heat flow profiles, we hypothesized that Pd-hydride formation can create a sufficiently energetic environment to facilitate the conversion of solid organic reactants with sufficiently low melting points and activation energies. To test this, the hydrogenolysis of BPE was chosen as a model reaction because BPE has a low melting point at 40 °C. The reaction yields toluene and phenol, is exothermic (Δ*H*_rxn_ ∼ −89 kJ mol^−1^, eqn (S6)), and has been shown to reach full conversion from BPE to aromatic monomer products in the presence of Pd catalysts.^37–56^

**Fig. 2:**
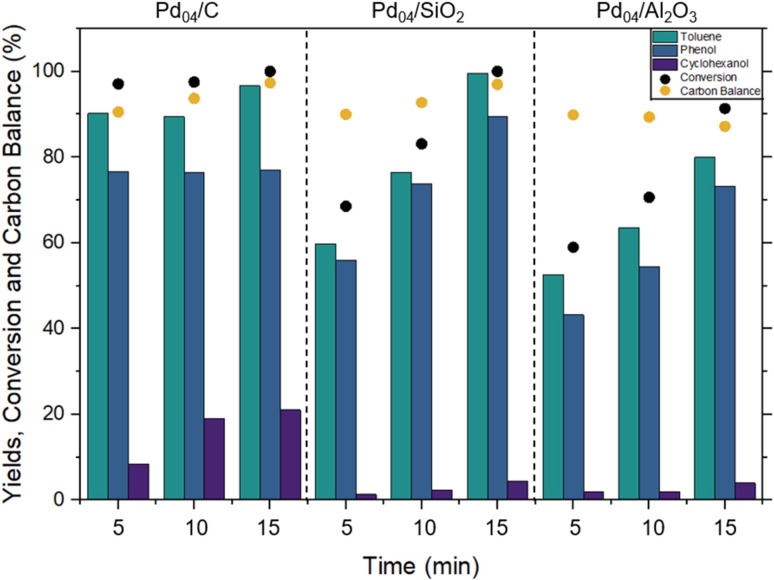
Toluene, phenol and cyclohexanol yields, BPE conversion and overall carbon balance (%) with respect to increasing reaction time (5–15 min, 3 Hz) at 30 sccm H_2_. For each experiment, 0.2 g of BPE was used in combination with 0.25 g of catalyst.

Without BPE present, Pd-H formation appeared to be nearly completed after 5 min based on TGA/DSC observations. Adding BPE to the system as a feedstock resulted in simultaneous Pd-H formation and consumption. The results for varied milling times (5–15 min) are shown in Fig. 2. After 5 min of H_2_ flow, Pd_04_/C achieved 97% conversion, while Pd_04_/SiO_2_ and Pd_04_/Al_2_O_3_ allowed for 70% and 60% conversion, respectively. After 15 min of H_2_ flow, conversion reached 100% for Pd_04_/C and Pd_04_/SiO_2_ and 91% for Pd_04_/Al_2_O_3_. This suggested that 15 min of H_2_ exposure was necessary to complete BPE conversion. Following H_2_ exposure for 5–15 min, the vessel was purged with N_2_ for 1 h to collect toluene as a volatile product in a downstream methanol trap. This increased the volatile toluene yields from below 10%, to 40–60% after 60 min of N_2_ purging. This delayed product collection suggests that the transport of toluene to the downstream collector was kinetically limiting. Without external heat application, the desorption of toluene from the catalysts surface close to STP conditions inherently slowed the elution process. Toluene desorption from Pd_04_/C appeared to be slower, due to van der Waals interactions between the carbonaceous products and the carbon support. Thus, toluene collected in the methanol trap contribute a maximum of 40% to the yield, while about 60% was observed for Pd_04_/SiO_2_ and Pd_04_/Al_2_O_3_. The combined toluene yield from collection of volatiles and washing the spent catalysts is reported as a cumulative yield in Fig. 2.

The experimental results of BPE hydrogenolysis showed strong agreement with the heat release observed by TGA/DSC. For catalysts with a higher heat release resulting from Pd-H formation, a higher rate was observed for the hydrogenolysis reaction and therefore greater conversion of the BPE dimer to toluene and phenol. Specifically, Pd_04_/C exhibited the highest reactivity, aligning with its greater heat flow, while Pd_04_/SiO_2_ and Pd_04_/Al_2_O_3_ showed lower conversion rates, reflecting their comparatively lower exothermic output. These findings suggest that the intrinsic heat released during Pd-hydride formation plays a crucial role in reaction facilitation, with catalysts generating higher heat flow achieving greater ether cleavage efficiency.

The X-ray diffractograms (XRD) showed marginal decreases for full width at half maximum (FWHM) values, indicating that the Pd particles were resistant to sintering under these reactive conditions when exposed to both H_2_ and BPE (Table S1 and Fig. S3). X-ray absorption spectroscopy (XAS) was used to determine shifts in Pd oxidation state, and the extended X-ray absorption fine structure (EXAFS) region was used to derive the Fourier transformed R-space for Pd_04_/C, Pd_04_/SiO_2_ and Pd_04_/Al_2_O_3_. At the experimental Pd-K edge, increases in Pd-Pd peak intensity at 2.5 Å were observed, accompanied by a slight rightward shift, indicating Pd-hydride formation and lattice expansion occured (Fig. S4 and S5).^33,35,56,74–77^ The most significant increase was observed after 5 min of H_2_ exposure. Increasing time thereafter resulted in minor increases in peak intensity at 2.5 Å, indicating that the majority of Pd particle reduction occurred in the first few minutes of H_2_ exposure, which is in good agreement with the TGA/DSC results (Fig. 1).

In addition to XRD and XAS, surface area was investigated using N_2_ physisorption, while changes in Pd particle size and morphological changes were investigated using TEM and scanning electron microscopy (SEM), respectively. The surface area (Fig. S6) further reflected the trend demonstrated for BPE hydrogenolysis activity, where Pd_04_/C had the highest surface area (965 m^2^ g^−1^) and demonstrated the highest activity, while Pd_04_/Al_2_O_3_ had the lowest surface area (106 m^2^ g^−1^), demonstrating the lowest activity; the surface area for Pd_04_/SiO_2_ fell in between these two values at 214 m^2^ g^−1^. The strong correlation between surface area and activity might indicate that the BPE hydrogenolysis becomes more efficient when the melted reactant is spread over a larger surface area, possibly due to more efficient H_2_ transport. TEM images (Fig. S7–S9) showed minor changes to Pd particle size (<1 nm) after milling, confirming that dispersion remained high for all three catalysts and heavy sintering did not occur. SEM images (Fig. S7–S9) also demonstrated an unchanging morphology of the catalytic supports, wherein the original structure was maintained post-reaction due to the low impact of the reaction.

**Fig. 3:**
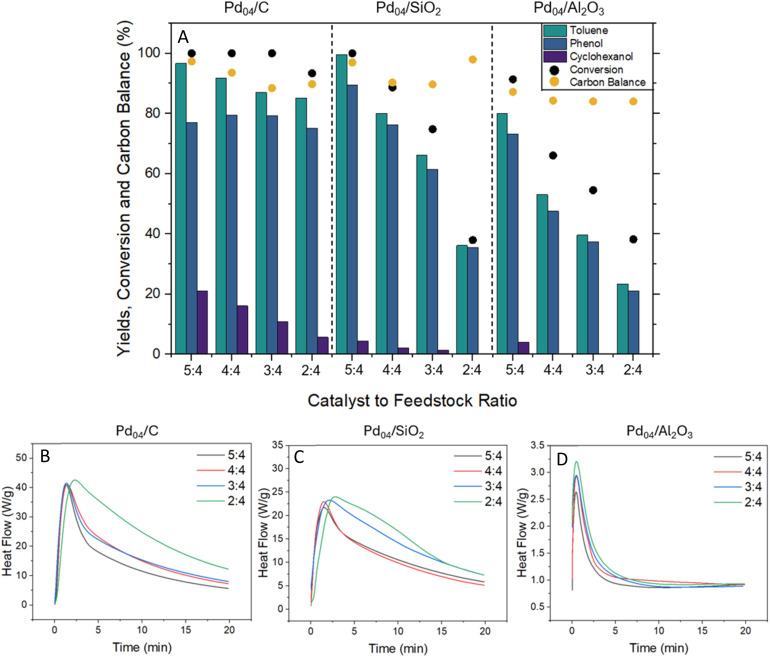
Toluene, phenol and cyclohexanol yields, BPE conversion and overall carbon balance (%) with respect to changing catalyst loading (0.10–0.25 g) (A). Time was held at 15 min with a mixing frequency of 3 Hz. H_2_ flow was held at 30 sccm while BPE was held at 0.20 g. Changes in heat flow (W g^−1^) for the altered catalyst-to-feedstock ratios are depicted in (B), (C), and (D) to demonstrate that reducing catalyst loading results in increased heat flow. TGA/DSC experimental conditions are explicitly described in S1.4.3.

Following these findings, the catalyst to feedstock ratio was adjusted to observe how decreasing Pd loading affected heat flow generation and the efficacy of triggering the hydrogenolysis reaction. The standard reaction conditions used 0.25 g of supported Pd catalyst, which was reduced to 0.20 g, 0.15 g and 0.10 g (Fig. 3A) while BPE was held constant at 0.20 g. At 0.10 g of catalyst loading, Pd_04_/C conversion fell to 93% while Pd_04_/SiO_2_ and Pd_04_/Al_2_O_3_ fell to 38% conversion. The reduced catalyst to feedstock ratios were further investigated using TGA/DSC (S1.4.3), in which heat flow (in W g^−1^) directly reflected the conversion of BPE observed in these experiments (Fig. 3B–D). These experiments were performed with the same catalyst/feedstock ratios as the reactions in the milling vessel. A mixture of 10 mg, 8 mg, 6 mg or 4 mg of catalyst was used in combination with 8 mg of BPE for the experiments in Fig. 3B–D. H_2_ gas flow was set to 10 mL min^−1^, and the experiments were conducted for 20 min without any external heat application.

For Pd_04_/C, conversion remained at 100% for catalyst weight loadings between 0.25–0.15 g, however, once the catalyst loading was reduced to 0.10 g, the reaction was incomplete and resulted in 93% conversion. The normalized heat flow slightly increased from 40.9 W g^−1^ to 41.4 W g^−1^ as the catalyst to feedstock ratio was decreased from 5 : 4 to 3 : 4, and further increased to 42.5 W g^−1^ at 2 : 4 loading. The same behavior was observed for Pd_04_/SiO_2_ and Pd_04_/Al_2_O_3_, and increased normalized heat flow occurred with each decrease in catalyst loading. The heat flow over Pd_04_/SiO_2_ increased from 21.7 to 24.0 W g^−1^, while that over Pd_04_/Al_2_O_3_ increased from 2.6 to 3.2 W g^−1^ as the catalyst to feedstock ratio was decreased from 5 : 4 to 2 : 4. This increase in normalized heat flow with respect to weight is expected, as the total weight of the catalyst and feedstock mixture is reduced. With a lower total mass of material, the overall heat capacity is lowered, and a greater value is observed for a specific amount of heat released. Congruently, the unnormalized heat flow decreased as the catalyst loading decreased for all three catalysts (Fig. S10). While Pd_04_/C and Pd_04_/SiO_2_ retained relatively greater heat flows when combined with BPE, the heat flow for Pd_04_/Al_2_O_3_ significantly decreased when compared to the heating of the fresh catalyst in Fig. 1. This is speculated to be related to surface area, as Pd_04_/C and Pd_04_/SiO_2_ had greater surface areas when compared to Pd_04_/Al_2_O_3_. With BPE present in the mixture, the reaction becomes transport limited, as BPE likely fills the pores of the catalyst support, leading to inaccessible Pd sites and ultimately reduced overall activity for Pd_04_/Al_2_O_3_. As available Pd sites are obstructed, Pd-hydride formation becomes hindered and consequently, heat flow is significantly lowered.

## Conclusion

The intrinsic exothermicity of Pd-hydride formation is sufficient to facilitate the cleavage of ether bonds in the lignin model dimer, BPE. For isolated catalysts, the majority of Pd-hydride formation occurs within the first 5 min of H_2_ exposure. BPE melts due to the released heat, which allows for intimate contact with the metal hydride sites, where the exothermic hydrogenolysis reaction can occur. Due to the mild nature of the reaction environment, with no external temperatures or pressures applied to the milling system, chemisorption of products on the catalyst supports was avoided, resulting in high carbon balances of 87–97% for the three catalysts under all reaction conditions. Based on the differences observed between these three catalysts, it can be concluded that the heat from Pd-hydride formation can be used as a substitute for an external energy source to trigger an exothermic reaction.

## Conflicts of interest

The authors declare no competing financial interest.

## Supplementary Material

SU-003-D5SU00574D-s001

## Data Availability

The data supporting this article have been included as part of the supplementary information (SI). Raw data files are available upon request to the authors. Supplementary information: analytical data including heat capacity correlation plots and calculations, XRD, XAS (XANES edge and R-space), N_2_ Physisorption results for surface area and pore diameter, SEM and TEM images, and TGA/DSC results. See DOI: https://doi.org/10.1039/d5su00574d.
