# Supplementary material for: Triggering hydrogenolysis of the lignin model compound benzyl phenyl ether using the intrinsic exothermicity of Pd-hydride formation
Source: RSC Sustain. 2025 Oct 8;3(11):5388–93. doi: 10.1039/d5su00574d (PMC12517400; doi:10.1039/d5su00574d)
Supplement: SU-003-D5SU00574D-s001 [file SU-003-D5SU00574D-s001.pdf]

This journal is © The Royal Society of Chemistry 2025

Triggering Hydrogenolysis of the Lignin Model Compound Benzyl Phenyl Ether (BPE) Using the Intrinsic Exothermicity of Pd-Hydride Formation

Erin V. Phillips[a], Marta C. Hatzell[b,c], Carsten Sievers[a,c*]

[a]School of Chemistry and Biochemistry, Georgia Institute of Technology, Atlanta, GA 30332, United States

[b]School of Chemical & Biomolecular Engineering, Georgia Institute of Technology, Atlanta, GA 30332, United States

[c]George W. Woodruff School of Mechanical Engineering, Atlanta, GA 30318, United States

Email: carsten.sievers@chbe.gatech.edu

Number of Pages: 18

Number of Figures: 10

Number of Tables: 1

Number of Images: 12

Number of Equations: 6

Section S1: Materials and Methods

S1.1 Benzyl Phenyl Ether as an α-O-4 Model Compound and Chemicals

S1.1.1 Chemicals

Benzyl phenyl ether (BPE, 97%) was purchased from Alfa Aesar. Decane (>99.0%) was obtained from Sigma Aldrich, along with Pd on carbon and Pd on Al_2O_3. Pd on SiO_2 was purchased from STREM Chemicals, pre-reduced. The three catalysts were commercially advertised as having a 5 wt% loading, but ICP was conducted to verify the actual Pd loading. Methanol (ACS grade) was purchased from VWR while H_2 and N_2 (UHP, Grade 5) were purchased from Airgas.

S1.2 Mechanocatalytic Reaction Setup

S1.2.1 Mechanocatalytic Hydrogenolysis Reactions

The mechanocatalytic reactions were conducted using a Retsch MM400 vibrational ball mill. A 25 mL stainless steel vessel was used to hold the feedstock and catalyst during the reactions, which was adapted with two outlets to allow for continuous gas flow. All reactions were run at a frequency of 3 Hz. A 25 mL methanol trap was placed at the end of the effluent line to collect all volatile products from the reactor. The line, also composed of stainless steel, measured 1/8 inch in diameter and was wrapped in heating tape to ~ 150°C to ensure that all volatile products eluted through the line. The lines directly connected to the inlet and outlet of the vessel were made of Teflon and measured 1/8 inch in diameter.

For each experiment, 0.2000 ± 0.0005 g of BPE and 0.2500 ± 0.0005 g Pd catalyst were added to the vessel, while ~ 0.030 – 0.050 g of decane was added to the methanol trap as an internal

standard. Once the vessel was closed and connected to the gas flow lines, the reaction began by milling at 3 Hz under N_2 at 15 sccm for 15 minutes, to purge the vessel of atmospheric air, followed by purging for 5 - 15 min of H_2 flow at 30 sccm and 3 Hz. H_2 was flowed at ~0.00134 mL/min (30 sccm), therefore, after 15 min, a total of 0.02012 mol of H_2 was provided to the reaction system. By comparison, 5 min at 30 sccm provided 0.00671 mol H_2 while 10 min at 30 sccm provided 0.0134 mol H_2. Because 0.2 g of BPE (0.001086 mol) was used with 0.25 g catalyst at 4% weight loading (0.000094 mol Pd), H_2 was provided in excess to complete both Pd-H formation and BPE hydrogenolysis. After the reaction, 60 min of stand-still N_2 purging was conducted, to make sure that all volatile products had been removed. Gas flow was controlled using an Omega mass flow controller (MFC).

To measure toluene production evolution, ~0.5 mL aliquot samples were taken from the methanol trap after the first 5, 10 or 15 min of H_2 flow, and after 60 min of N_2 purging had been completed. After reaction completion, the contents of the vessel were collected and measured. The collected catalyst-product mixture was diluted with 15 – 20 mL of methanol, and 0.030 - 0.050 g of decane was once again added as an internal standard. The mixture was sonicated for 30 mins and filtered afterwards to obtain a pure liquid sample of the diluted non-volatile products for product quantification using GC-FID. All experiments were conducted in triplicate for consistency to ensure that the standard deviations for the averaged results were below ± 5%.

S1.2.2 Adjusted Catalyst to Feedstock Ratio Experiments

For adjusted catalyst to feedstock ratio milling experiments, 0.2000 ± 0.0005 g of BPE and 0.1000 - 0.2500 ± 0.0005 g Pd catalyst were added to the vessel, while ~ 0.030 – 0.050 g of decane was added to the methanol trap as an internal standard. The vessel was placed into the milling setup, and the same procedure followed as outlined above; all experiments for this section exposed the catalyst/feedstock mixture to 15 min of H_2 flow at 30 sccm. All volatile products were again collected via methanol trap, while non-volatile products were dissolved in methanol with decane and sonicated for 30 mins.

S1.3 Product Analysis: Yields, Conversion and Carbon Balance

S1.3.1 Gas Chromatography with Flame Ionization Detection (GC-FID)

All product yields were determined using a Varian 450-GC equipped with an FID detector and PolyArc methanizer reactor (Activated Research Company). A fused silica column was used (Supelco, SPB-1, 60 m x 0.25 mm x 1.0 µm) within the GC for product separation. As previously mentioned, decane was used as an internal standard, so no calibration was required when used in tandem with the PolyArc methanizer. Example calculations are shown below (Eq S1 and S2):

$$n_{product} = \frac{A_{product}}{A_{Standard}} * \frac{C_{Standard}}{C_{Product}} * \frac{m_{Standard}}{MW_{Standard}} \quad \text{(Eq S1)}$$

$$Yield_{Product} = \frac{n_{Product}}{n_{BPE\ Initial}} * 100\% \quad \text{(Eq S2)}$$

$$A = GC - FID\ Peak\ Area$$

$$C = \#\ of\ carbons\ within\ the\ molecular\ structure$$

S1.4 Microscopic Catalyst Characterization Before and After Milling

S1.4.1 Inductively Coupled Plasma (ICP) Analysis

To prep the samples for ICP, the unmilled catalysts were first weighed and pre-digested in a 50 mL beaker and placed on a hot plate. 5 mL of concentrated nitric acid (Trace metal Grade from Fisher Chemical, UN2031) was added to the beaker. The mixture was heated for 30 mins, then 2.5 mL of concentrated HCl (Trace metal grade from Fisher Chemical) was added. The mixture was brought to a slight boil for an additional 30 mins, then allowed to cool to room temperature. Afterwards, the mixture was filtered through 40 ashless Whatman filter paper and washed with DI water. The ICP was calibrated with Pd, Si, and Al, generating a four-point calibration curve for each element of interest. The prepared sample was injected into the ICP, and the target elements were quantified based on the calibration.

S1.4.2 Transmission Electron Microscopy (TEM)

A FEI Tecnai F30 was used to obtain TEM images at an operating kV of 300. The instrument was equipped with a thermally assisted field emission (TFE) gun, and all images were converted and analyzed using a Gatan GIF system (Tridiem 863 UHS). For preparation, approximately 5 mg of the samples were sonicated in 1 mL of ethanol and dispersed onto a holey carbon – copper 200 mesh grid. Initial particle sizes were calculated using the TEM image below (Figure S8A, S9A and S10A) by taking an average diameter of 90 particles. ImageJ software was used for the analysis. Based on the particle size determined by TEM, the following equation (Eq S3) was used to determine the % dispersion of the particles:

$$D\% = 6 * \frac{(^{v_m}/_{a_m})}{d_{Va}} \quad \text{(Eq S3)}$$

Here, v_m is defined as the volume occupied by an atom in the bulk of metal, where the MW of Pd is divided by the density of Pd and multiplied by Avogadro's number to give the constant value:

$$v_m = \frac{Pd_{MW}}{Pd_{Density} * AGs\ \#} = \frac{106.4\frac{g}{mol}}{12.03\frac{g}{cm^3} * 6.022E23}\ 1.4632\ \text{E-23 cm}^3\text{/atom}$$

The variable $a_m$is the atomic cross-sectional area where 0.0787 nm^2/Pd atom is a defined, non-changing value and d_{Va} is the particle size.

S1.4.3 Thermogravimetric Analysis with Differential Scanning Calorimetry (TGA/DCS)

TGA/DSC was completed using an SDT Q600 V20.9 Build 20. Approximately 7.5 mg of fresh catalysts were used for Figure 1 experiments, while a catalyst/feedstock mixture of 10 mg, 8 mg, 6 mg or 4 mg of catalyst was used in combination with 8 mg of BPE for the experiment in Figure 3. H_2 gas flow was set to 10 mL/min, and the experiments were conducted for 20 min. A heating gradient was not applied to the TGA program and all changes in temperature were a direct result of the exothermicity of palladium hydride formation.

S1.4.4 X-Ray Diffraction (XRD)

XRD was performed using a Rigaku Miniflex Powder XRD system. A CuKα x-ray source was used at a wavelength of λ = 1.54 Å. A current of 40 mV was applied while the generator operated at a voltage of 15 kV. The spectra were measured from 2θ = 20° to 70° with a step size of 0.01° and a scan speed of 10° per minute.

S1.4.5 X-Ray Adsorption Spectroscopy (XAS)

X-ray absorption spectroscopy data was collected at the SSRL Spectroscopy beamline (9-3) of the SLAC National Accelerator Laboratory, operated by Stanford University in connection with the U.S. Department of Energy (DOE). The samples were pressed into wafers by diluting the catalysts with cellulose in a 1:4 ratio. The energy was calibrated using a Pd foil, measured together with the samples, and the edge for metal Pd was set to 24350 kV. To process the data, the Demeter package was used. Four scans were collected and averaged for each sample to improve data quality.

S1.5 Macroscopic Catalyst Characterization Before and After Milling

S1.5.1 N_2 Physisorption

Surface area and pore volume were measured using N_2 physisorption coupled with Brunauer–Emmett–Teller (BET) analysis. The analysis was conducted on a Micromeritics ASAP 2020 Accelerated Surface Area and Porosimetry System. The sample (0.200 g) was degassed to 10 mmHg and held under vacuum for 120 mins before backfilling. The sample was then shifted to the analysis port where a standard N_2 physisorption method was used to complete the desorption.

S1.5.2 Scanning Electron Microscopy (SEM)

SEM was conducted using a Thermo Axia Variable Pressure Model with a thermionic tungsten filament source. The samples were prepared on carbon tape and were analyzed under high vacuum at 3.00 kV - 10.00 kV at a working distance of 9.5 to 11.0 mm. An Everhart-Thornley detector was used for SEM imaging, which ranged from 50 μm to 100 μm scales.

Section S2: Calculations for ΔH of Pd-H Formation, ΔT Based on Catalytic Support Heat Capacity, and ΔH of BPE Cleavage

S2.1 ΔH of Pd-H Formation

For the three catalysts, the exact weight percent was 4.07% for Pd_{04}/C, 3.84% for Pd_{04}/SiO_2 and 3.62% for Pd_{04}/Al_2O_3. Jewell et al. investigated hydrogen adsorption on Pd(111), Pd(110), and Pd(100), identifying multiple adsorption sites, including face-centered cubic (fcc), hexagonal close-packed (hcp), bridge sites, and top sites.[1, 2] Hydrogen atoms most commonly occupy the octahedral interstitial sites of the fcc metal lattice, where the associated exothermic ΔE_{ads} for Pd hydride formation ranges from -51 kJ/mol to -111 kJ/mol for site adsorption on the (111) plane under ambient conditions.[1, 3] Literature has shown that the heat of formation for Pd-H ranges from -51 kJ/mol to -111 kJ/mol at the (111) Pd site, so -70 kJ/mol was used as a conservative estimate.

$$\Delta Hrxn = \left(\frac{Catalyst\ Weight*Pd\ Weight\ \%\ Loading}{MW\ of\ Pd}\right) * Heat\ of\ Formation\ of\ Pd - H \quad \text{(Eq S4)}$$

Sample calculation for Pd_{04}/C:

$$\Delta Hrxn = \left(\frac{250\ mg * 0.0407}{106.42\ g/mol}\right) * -70\frac{kJ}{mol} = -6.73\ J$$

These values are plotted against the weight loading of each catalyst for various amounts of catalysts ranging from 0.10 g to 0.25 g in Figure S1.

S2.2 ΔT Based on Catalytic Support Heat Capacity

The heat capacity of carbon is 0.71 J/g*K, silica is 0.70 J/g*K and alumina is 0.90 J/g*k.

$$\Delta T = -\left(\frac{\Delta Hrxn}{Support\ Heat\ Capacity*Catalyst\ Weight}\right) * Pd{:}\,H\ Formation\ Ratio \quad \text{(Eq S5)}$$

Sample calculation for Pd_{04}/C:

$$\Delta T = -\left(\frac{-6.73\ J}{0.71\frac{J}{g*K} * 0.25g}\right) * \frac{3}{2} = 57^{o}C$$

S2.3 ΔH of BPE Cleavage to Toluene and Phenol

$$\Delta Hrxn = (BDE\ Bonds\ Broken) - (BDE\ Bonds\ Formed)$$

$$\Delta Hrxn = (BDE_{H-H} + BDE_{C-O}) - (BDE_{O-H} + BDE_{C-H}) \quad \text{(Eq S6)}$$

$$\Delta Hrxn = \left(435.8\frac{kJ}{mol} + 218.0\frac{kJ}{mol}\right) - \left(368.2\frac{kJ}{mol} + 375.5\frac{kJ}{mol}\right) = -89.9\ kJ/mol$$

Section S3: Supplementary Results

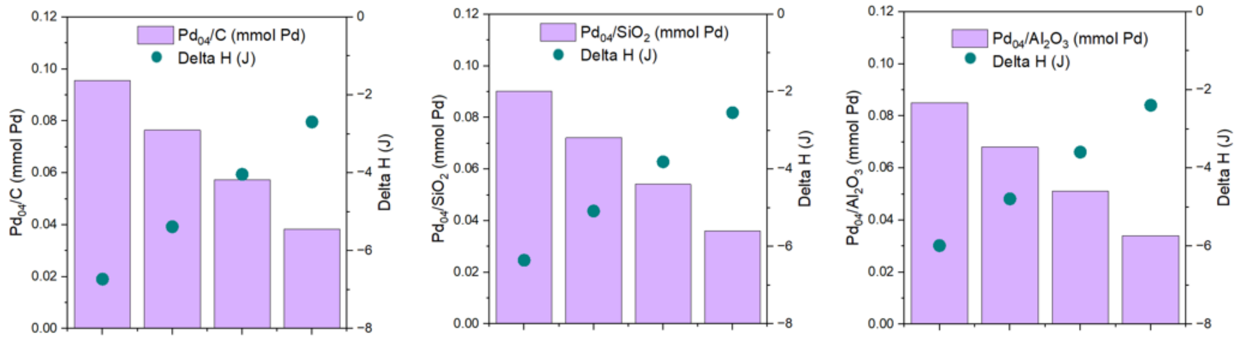

Figure S1: Delta H of Pd hydride formation (J) plotted in correlation to the amount of Pd wt% loading (mmol) for 0.25 g, 0.20 g, 0.15 g and 10 g samples of Pd_{04}/C, Pd_{04}/SiO_2 and Pd_{04}/Al_2O_3.

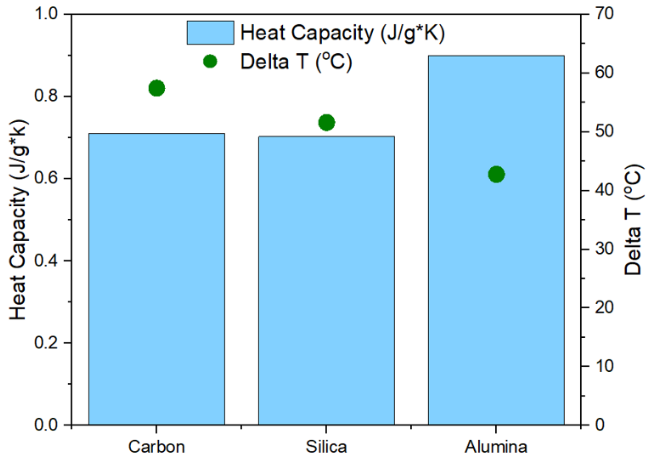

Figure S2: Heat capacity of the carbon, silica and alumina catalyst supports (J/g*K) plotted against the change in Delta T (°C) of the catalysts, based on Delta H (J) for Pd wt % loading in Figure S1 for 0.25 g samples of Pd_{04}/C, Pd_{04}/SiO_2 and Pd_{04}/Al_2O_3.

Table S1: FWHM values for Pd_{04}/C, Pd_{04}/SiO_2 and Pd_{04}/Al_2O_3 after 5 min, 10 min and 15 min of milling correlating to the main parent peak at 40° for the Pd (111) crystallite structure.

FWHM at °2θ	*Pd_{04}/C*	*Pd_{04}/SiO_2*	*Pd_{04}/Al_2O_3*
Fresh	4.57	1.13	1.98
5 min	2.57	1.06	1.86
10 min	2.62	1.09	1.76
15 min	2.67	1.06	1.68

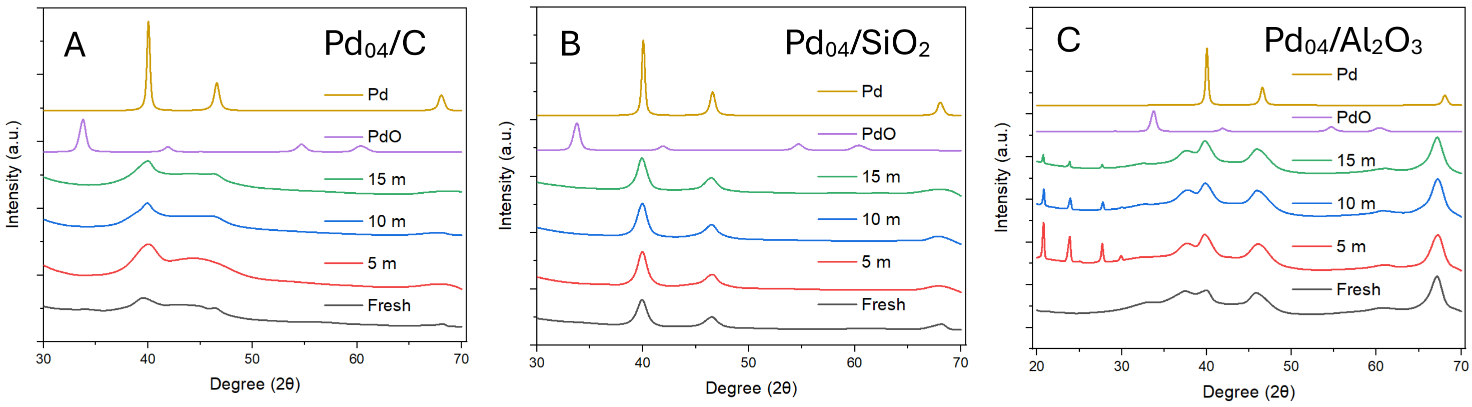

Figure S3: X-ray diffractograms for various reaction time experiments for Pd_{04}/C (A), Pd_{04}/SiO_2 (B) and Pd_{04}/Al_2O_3 (C) from 20° to 70° (2θ).

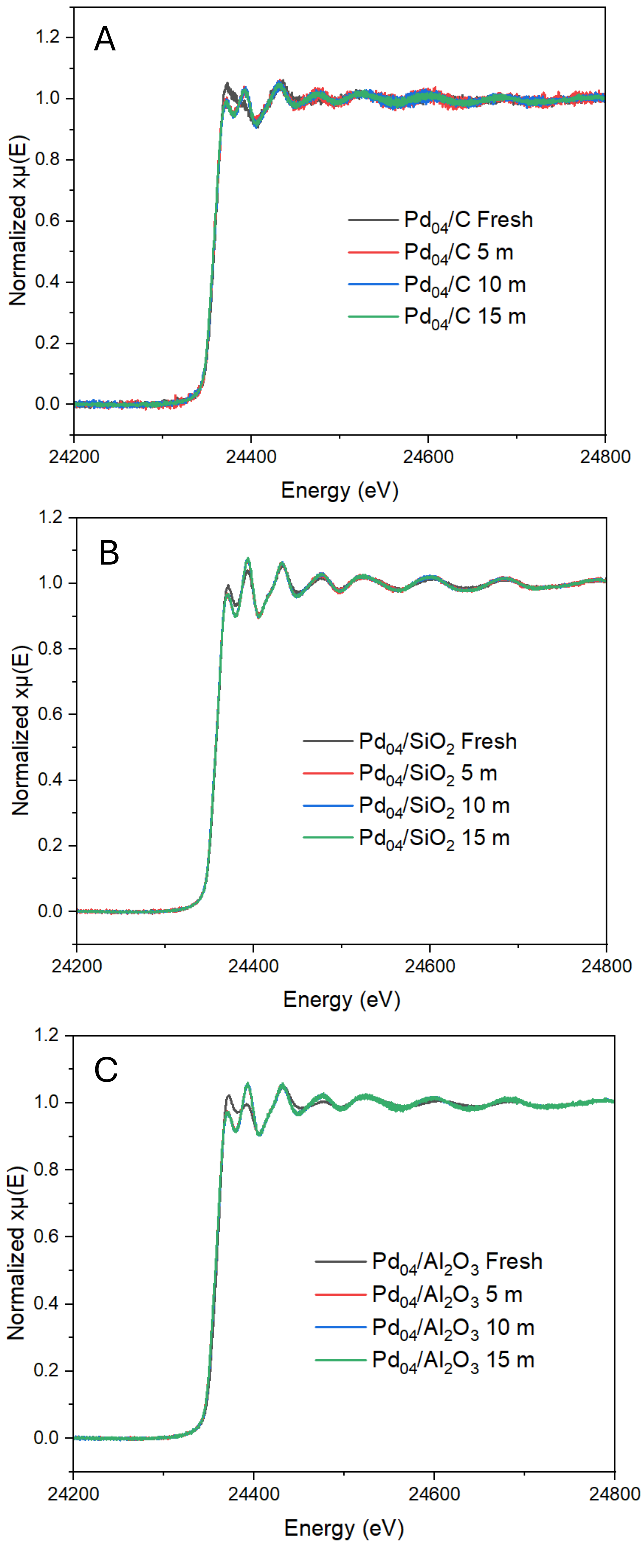

Figure S4: XAS spectra of the experimental Pd K-edge for Pd_{04}/C (A), Pd_{04}/SiO_2 (B) and Pd_{04}/Al_2O_3 (C) for various reaction times.

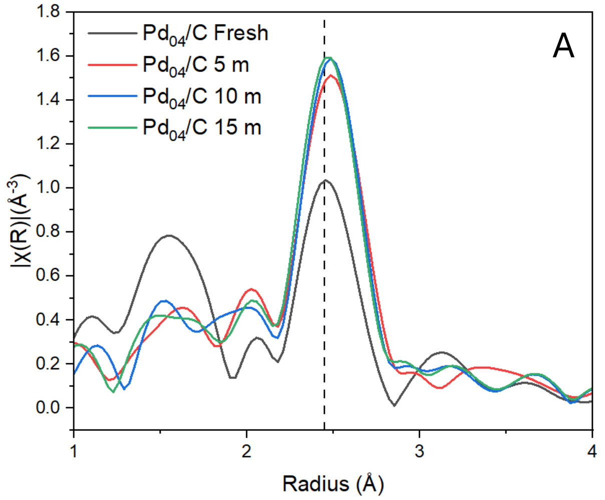

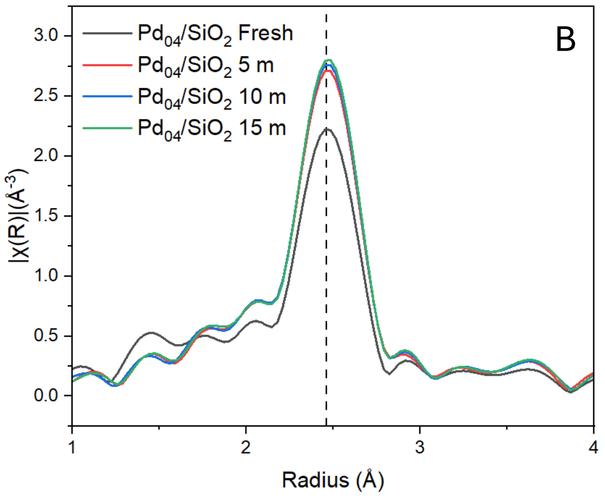

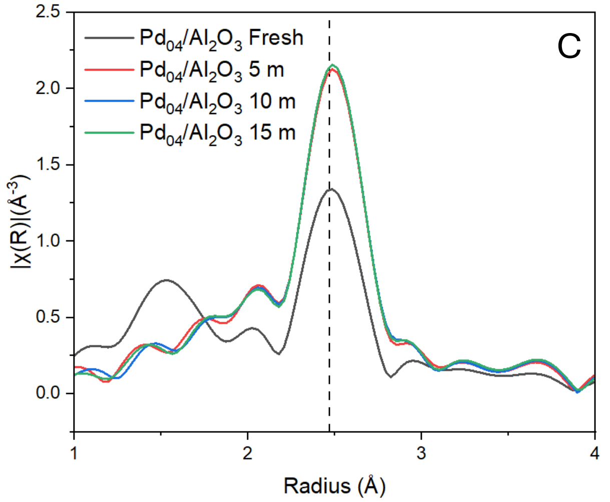

Figure S5: Fourier transformed R-space of the experimental Pd K-edge for Pd_{04}/C (A), Pd_{04}/SiO_2 (B) and Pd_{04}/Al_2O_3 (C) for various reaction times.

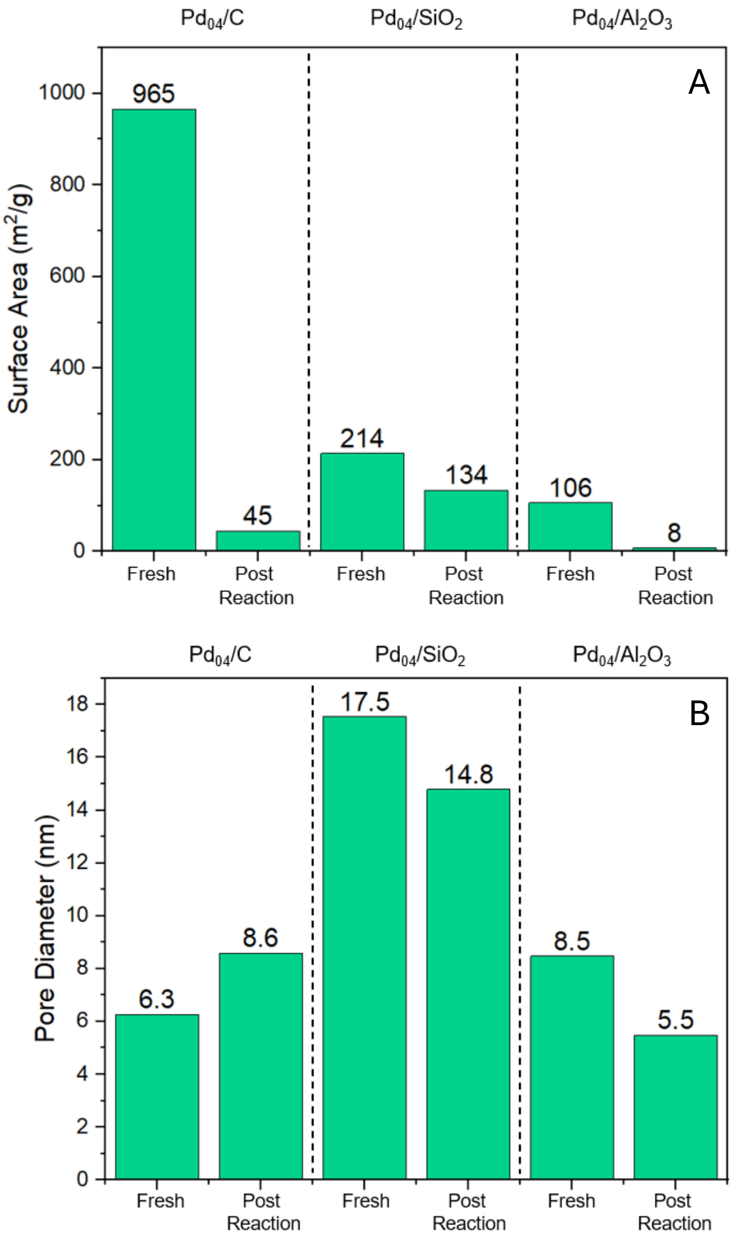

Figure S6: (A) Surface area (m^2/g) and (B) pore diameter (nm) for Pd_{04}/C, Pd_{04}/SiO_2 and Pd_{04}/Al_2O_3 before and after milling at 3 Hz for 15 min with H_2 flow set at 30 sccm.

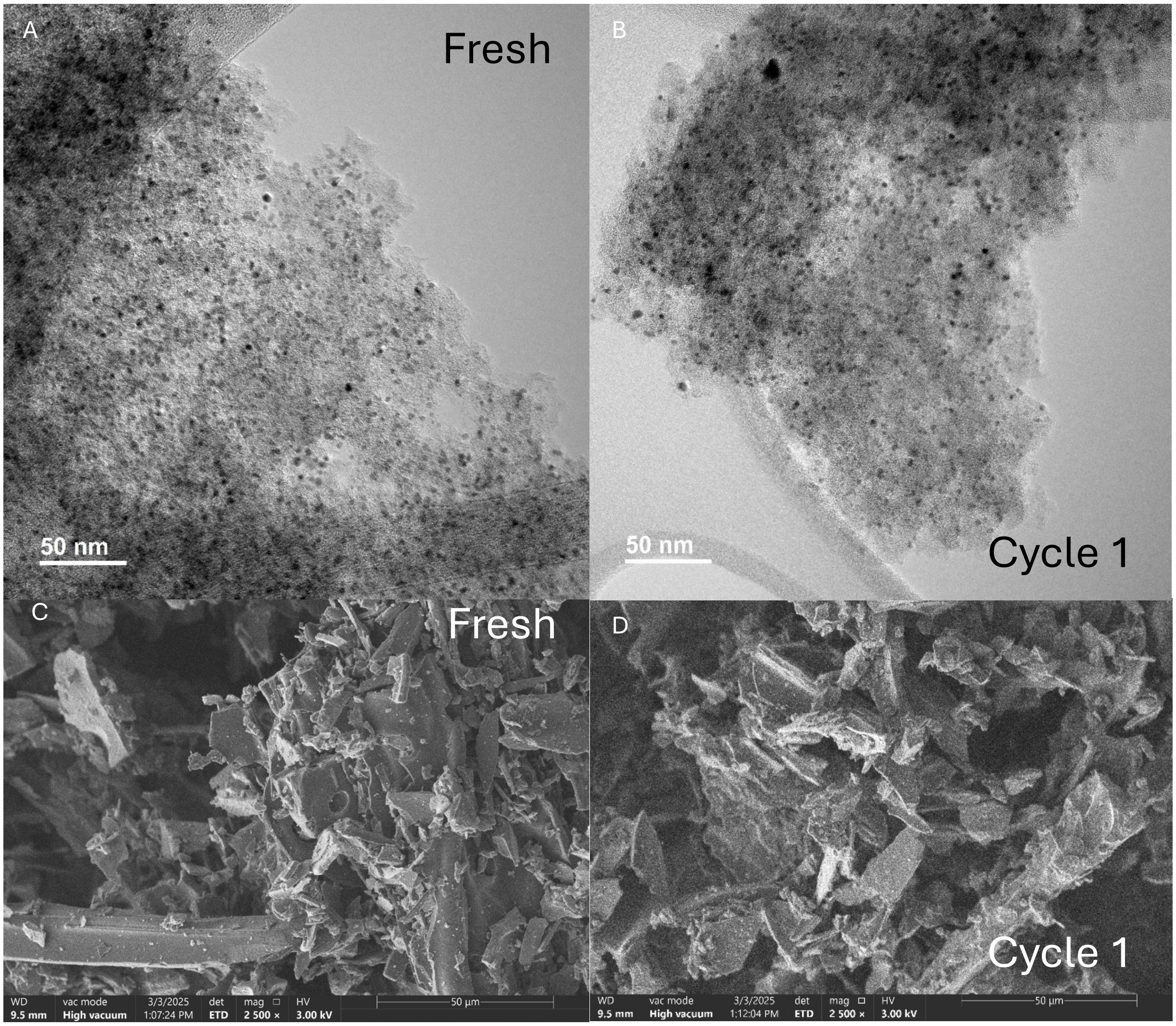

Figure S7: TEM (A and B) and SEM (C and D) images of Pd_{04}/C before and after BPE hydrogenolysis (15 min, 30 sccm H_2). TEM images are shown at a 50 nm scale while SEM images are shown at a 50 µm scale.

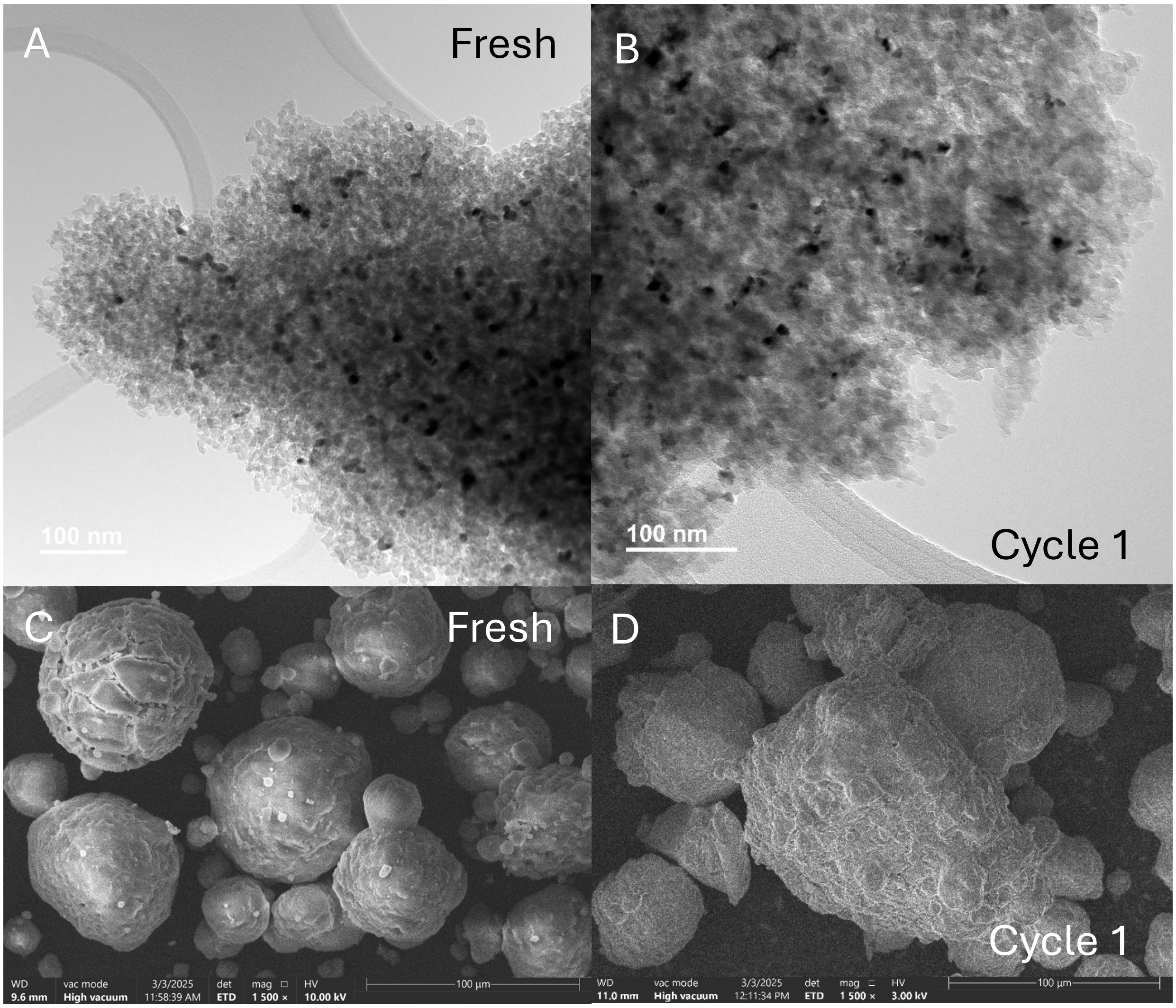

Figure S8: TEM (A and B) and SEM (C and D) images of Pd_{04}/SiO_2 before and after BPE hydrogenolysis (15 min, 30 sccm H_2). TEM images are shown at a 100 nm scale while SEM images are shown at a 100 µm scale.

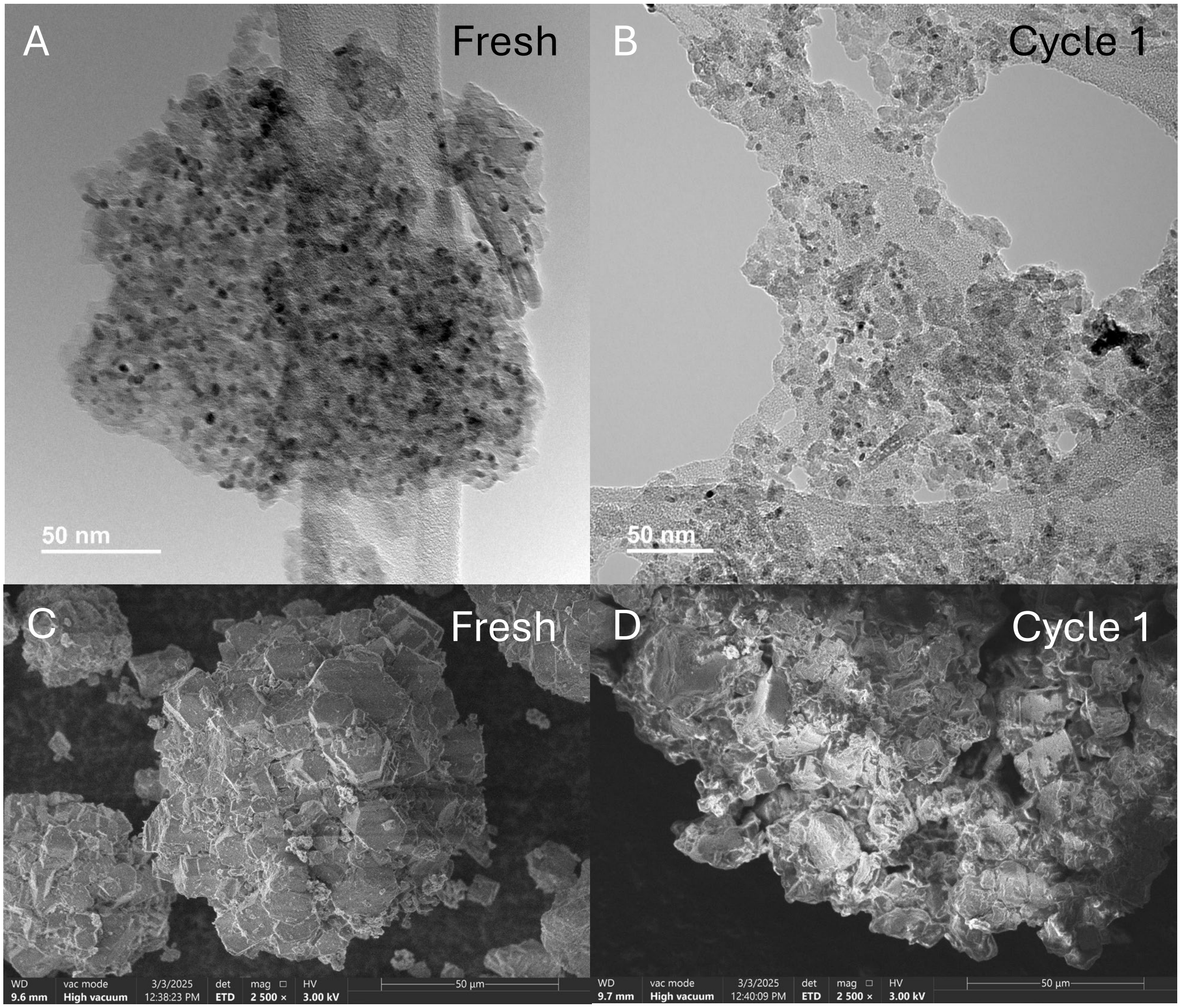

Figure S9: TEM (A and B) and SEM (C and D) images of Pd_{04}/Al_2O_3 before and BPE hydrogenolysis (15 min, 30 sccm H_2). TEM images are shown at a 50 nm scale while SEM images are shown at a 50 µm scale.

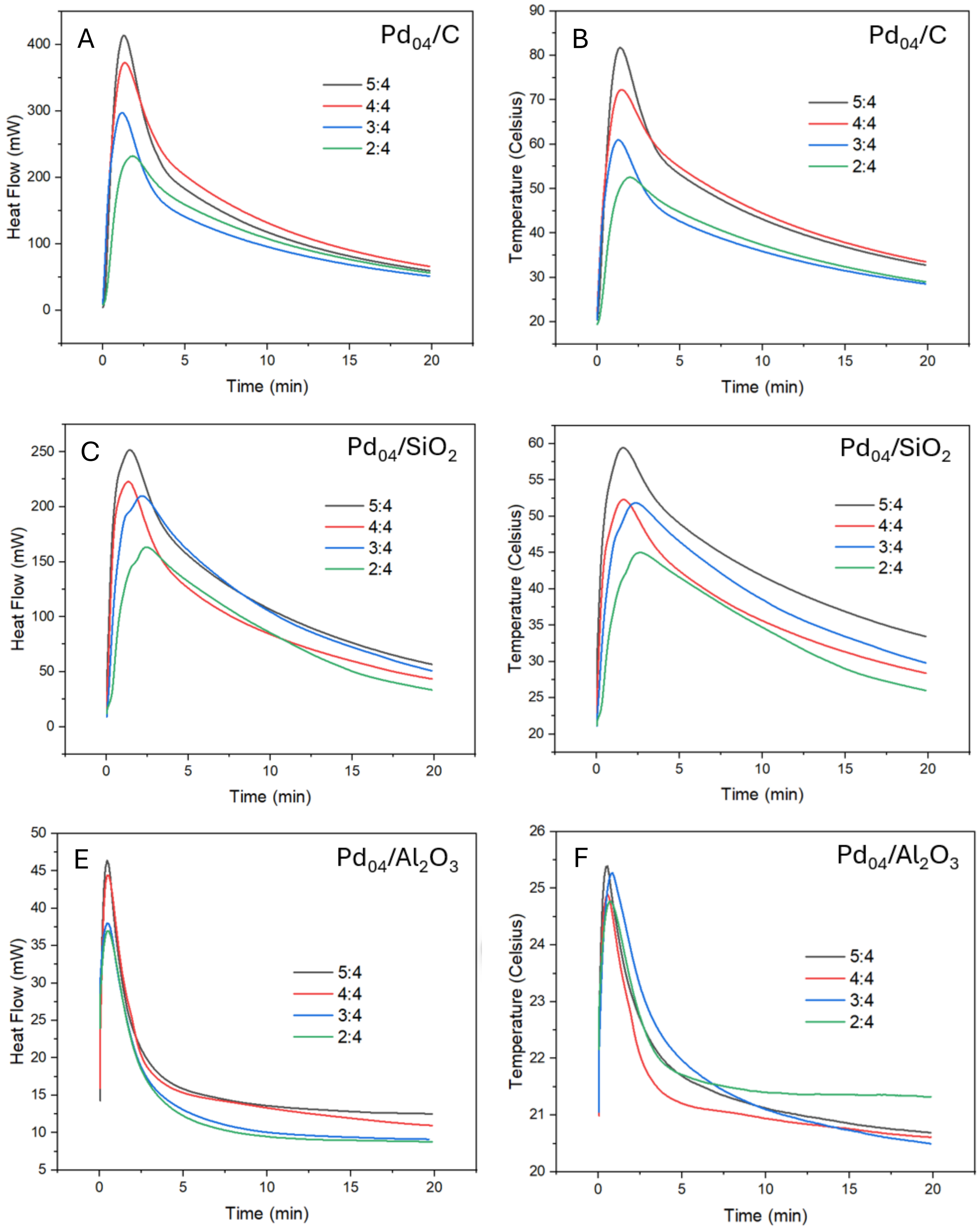

Figure S10: Thermogravimetric analysis for Pd_{04}/C (A and B), Pd_{04}/SiO_2 (C and D) and Pd_{04}/Al_2O_3 (E and F) for adjusted catalyst to feedstock ratios (0.10 g – 0.25 g). Change in temperature (ºC) and heat flow (mW) are shown.

References

(1) L. Jewell, and B. Davis, *Appl. Catal. A. Gen.,* 2006, **310**, 1-15.
(2) M. Johansson, E. Skúlason, G. Nielsen, S. Murphy, R. M. Nielsen and I. Chorkendorff, *Surf. Sci.,* 2010, **604**, 718-729.
(3) W. Dong, G. Kreese and J. Hafner, *J. Mol. Catal. A. Chem.,* 1997, **119**, 69-76.
